# Editorial: Mapping Psychopathology with fMRI and Effective Connectivity Analysis

**DOI:** 10.3389/fnhum.2017.00151

**Published:** 2017-03-31

**Authors:** Baojuan Li, Adeel Razi, Karl J. Friston

**Affiliations:** ^1^School of Biomedical Engineering, Fourth Military Medical UniversityXi'an, China; ^2^Athinoula A. Martinos Center for Biomedical Imaging, Department of Radiology, Massachusetts General Hospital and Harvard Medical SchoolBoston, MA, USA; ^3^The Wellcome Trust Centre for Neuroimaging, University College LondonLondon, UK; ^4^Department of Electronic Engineering, NED University of Engineering and TechnologyKarachi, Pakistan

**Keywords:** effective connectivity, fMRI, neuropsychiatric disorders, psychophysiological interactions, dynamic causal modeling, Granger causality

Distributed networks of interacting brain systems—rather than a single area—are usually involved in the execution of a specific cognitive task. Recent advances in neuroimaging techniques now allow us to see how these interacting brain regions are integrated and cooperate with each other to prosecute cognitive operations (Razi and Friston, 2016). Brain connectivity analyses based on electroencephalography (EEG), magnetoencephalography (MEG), and functional magnetic resonance imaging (fMRI) signals characterize neuronal responses in terms of how brain activity is induced by external stimuli and propagates among distributed brain regions, and thus may help answer key questions about functional brain architectures. The insights that brain connectivity analyses offer is also crucial for us to elucidate the neurobiological correlates underlying many neuropsychiatric disorders.

Functional connectivity and effective connectivity are generally used to measure functional integration in neuroimaging. The former examines (undirected) statistical dependencies (e.g., temporal correlations) between brain regions and has been extensively studied to characterize brain networks at rest. However, understanding the precise mechanisms mediating cognitive processes depend on directed information flow within brain networks. Thus, the current research topic focuses on mapping psychopathology with (directed) effective connectivity analysis, which models causal interactions among brain regions. We attempt to further improve current understanding of the neural mechanisms of major neuropsychiatric disorders by exploring how signals are transmitted differently from one region to another in healthy controls and patients. This comparison may help explain the pathophysiology and psychopathology seen in these disorders—at a network and possibly synaptic level.

In this research topic, we invited world-renowned experts to present their recent work that have utilized various models of directed connectivity; including psychophysiological interactions (PPI), Granger causality (GC), and dynamic causal modeling (DCM) to investigate directed connectivity in healthy subjects and patients with neuropsychiatric disorders. The papers in this research topic further our knowledge of the neurobiological mechanisms underlying neurological and psychiatric disorders. We will see that young people with OCD exhibit increased dorsal anterior cingulate cortex (dACC) modulatory effects during the performance of working memory tasks (Diwadkar et al.). There is also new evidence suggesting that neurodegenerative disorders like Parkinson's disease (Yan et al.) and Huntington's disease (Minkova et al.) are characterized by impaired causal interactions of the motor control system (Dowlati et al.) speaking to the interesting notion that age-dependent neural mechanisms may be important for understanding aberrant belief states associated with psychopathology. Interestingly, abnormalities in brain effective connectivity are also seen clearly in resting state in subjects with schizophrenia (Cui et al.), smoking addiction (Tang et al.), idiopathic generalized epilepsy (Wei et al.) and cocaine users (Ray et al.). In addition, it is exciting to see that effective connectivity analysis may furnish a new framework to understand fatigue and depression (Stephan et al.). We believe these findings have the potential to invigorate and advance our understanding of the neurobiological mechanisms of major neuropsychiatric disorders, thus improving the prevention, diagnosis and treatment of these disorders.

Looking into the future, we anticipate a shift toward new methods that can measure effective connectivity among specific cell types (e.g., lamina-specific connectivity). For example, methods that use detailed biophysical modeling based on canonical microcircuits and neural mass models for functional MRI data (Friston et al., in press) or that combine other modalities—like optogenetics—with fMRI (Bernal-Casas et al., 2017). These new methods will be very useful within the context of detecting early and selective abnormalities in specific cell types in various forms of dementia. For example, frontotemporal lobar degeneration (FTLD) has been previously shown to selectively target Von Economo neurons in fronto-insular regions (Seeley et al., 2006) and that pathogenic *huntingtin* protein selectively targets striatal spiny projection neurons (Ehrlich, 2012). More detailed and informed models of effective connectivity may also be very useful for furthering recent (and exciting) developments in understanding compensatory mechanisms in presymptomatic neurodegeneration that could potentially translate to the discovery of reliable neuronal biomarkers of disease progression (Klöppel et al., 2015). We envisage that computational modeling of dementia will usher a new era of functional integration research, by increasing our understanding of mechanisms by which molecular lesions engender specific meso and macro scale neural network damage that maps onto specific phenotypes (Gilson et al., 2016).

A complete understanding of complex (many-to-many) protein-network-phenotype mappings will be crucial for the early diagnosis and development of interventional therapies for slowing and preventing dementia. In psychiatry, computational modeling has already given a birth to the new field of computational psychiatry that constitutes a new paradigm for translational research and clinical decision making (Montague et al., 2012; Friston et al., 2014). The potential for developing new treatments for psychiatric illnesses that go beyond addressing symptoms is promising and any computational modeling that can precisely characterize aberrant connectivity may play a central role in predicting an individual's clinical trajectory (Stephan and Mathys, 2014; Friston, 2016).

## Author contributions

All authors listed, have made substantial, direct and intellectual contribution to the work, and approved it for publication.

### Conflict of interest statement

The authors declare that the research was conducted in the absence of any commercial or financial relationships that could be construed as a potential conflict of interest.
